# 3D visualization of microwave electric and magnetic fields by using a metasurface-based indicator

**DOI:** 10.1038/s41598-022-10073-7

**Published:** 2022-04-12

**Authors:** Zhirayr Baghdasaryan, Arsen Babajanyan, Henrik Parsamyan, Barry Friedman, Seungwan Kim, Jung-Ha Lee, Kiejin Lee

**Affiliations:** 1 Department of Physics, Sogang University, Seoul, 121-742 South Korea; 2 Department of Radiophysics, Yerevan State University, 0025 Yerevan, Armenia; 3 Department of Physics, Sam Houston State University, Huntsville, TX 77341 USA; 4 Department of Life Science, Sogang University, Seoul, 121-742 South Korea

**Keywords:** Nanoscale materials, Metals and alloys, Characterization and analytical techniques, Imaging techniques, Microscopy, Surface patterning

## Abstract

Visualizations of the microwave electric and magnetic near-field distributions of radio-frequency (RF) filters were performed using the technique of thermoelastic optical indicator microscopy (TEOIM). New optical indicators based on periodic dielectric-metal structures were designed for electric field visualization. Depending on the structure orientation, such metasurface-based indicators allow separately visualization of the *E*_x_ and *E*_y_ components of the in-plane electric field. Numerical simulations were conducted to examine the working principle of the designed indicator structures, and the results were compared to the experimental, showing good agreement. In addition, the 3D visualization of the microwave near-field distribution was built, to show the field intensity and distribution dependencies on the distance from the RF filter.

## Introduction

Microwave imaging techniques have been widely investigated and used for several applications. This broader class of imaging methods is a promising tool in biomedical diagnosis^1,2^, defect inspection^3–7^, material characterization^8–10^, semiconductor device design^11–14^, etc. In recent years, the focus on microwave imaging technology in medical applications has increased significantly because of its non-invasive and non-destructive diagnosis ability. In this area, these techniques are used to visualize malignant cells in the early stage, breast tumors, and brain tissue^1,15,16^. During the past few decades, different microwave imaging technology has developed to observe the structure of the electromagnetic field. One of the attractive methods is a scanning-probe microscope, where the probe moves along the sample surface by detecting the interaction between the sample and the radio-frequency (RF) field^13,14,17,18^. These microscopes can reach an extremely high spatial resolution in the range of nanoscale^19^. The scanning-probe methods have a disadvantage in the slow measured throughput and the complex experimental equipment limits their practical applications. Other existing imaging methods based on the metamaterial absorber provide fast measurement time with outstanding high resolution. Metasurface-based sensors are used to develop microwave cameras^20,21^. These cameras can reach a subwavelength spatial resolution, but on the other hand, the resolution of these sensors is limited by unit-cell size. There are also different optical ways to visualize microwave near-field (MWNF) distribution. The live electro-optic imaging technique is one of them^22–25^. The basic principle of this method is based on the Pockels effect, and a ZnTe electro-optic crystal is used as an electric field sensor. Since the Pockels effect is an electro-optic effect, it can visualize the microwave electric field distribution, and this system is suitable for performing the real-time measurement.

Thermoelastic optical-indicator microscopy (TEOIM) is another optical method for visualizing MWNF distribution. This paper presents the applications and methods of TEOIM, and the main focus is placed on a new type of optical indicators (OI), which are based on the metastructure. The previous publications of this TEOIM system include detailed information about the working principles and image processing methods^26^. Recently, it was shown that the TEOIM visualization system is applicable to a medical environment and is a promising tool for diagnosis for biological samples^27^. Simple indium-tin-oxide (ITO)-coated glasses are excellent indicators for a magnetic field visualization, but for electric field visualization, it is hard to find and fabricate indicators having high dielectric losses for visualizing the electric field. The current research presents an easy solution for this kind of indicators, and it is based on the periodic structure. For electric field visualization, the meander chain metasurface (MCM) was designed using ITO glasses. These designed metasurface-based indicators are able to visualize |*E*_x_| and |*E*_y_| components of the in-plane electric field separately.

Due to the experimental agility of TEOIM, imaging a MWNF distribution through the photoelastic effect is an attractive technique for electric and magnetic near-field visualization. The ability to visualize an electric or magnetic field depending on the OI properties is one of the benefits of the TEOIM. The experimental setup is straightforward to configure without any expensive or complex equipment. Moreover, it has been experimentally demonstrated that the TEOIM imaging system is capable of performing high-resolution MWNF images without spatial scanning. The fast measurement allows real condition experimental validation instead of simulations and enables the design of unique prototypes and commercial systems for various applications. In the future, with advanced indicators, the system will be widely applicable.

## Experimental setup

**Figure 1:**
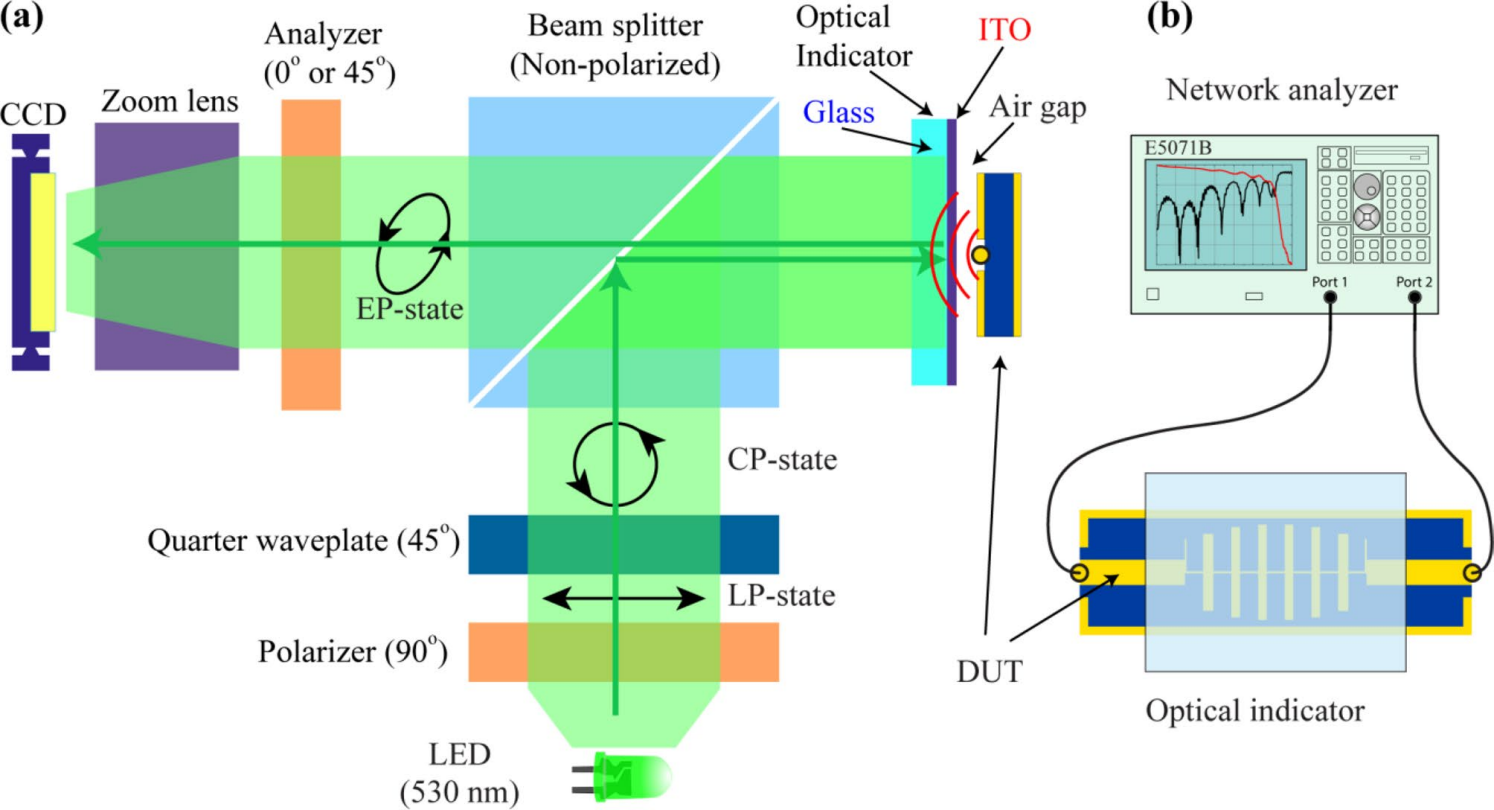
(**a**) Illustration of the visualization system. Successively, light passes through a linear polarizer and a quarter waveplate, consequently becoming circularly polarized. Probing light is directed to an OI fixed on a DUT. The light reflected from the OI passes through the analyzer (linear polarizer sheet), and the CCD camera registers the intensity of the light. (**b**) The configuration of the DUT connected with a network analyzer. The ITO was placed on the structure of the device with a small air gap.

Figure 1 shows (a) the schematic of the experimental setup of TEOIM and (b) measurement configuration of device under test (DUT). This technique is a polarized light microscope system where commercially available ITO-coated glass (Eagle XG, 0.7 mm) was used as an OI. The light from the LED ($\lambda =530\, \text{nm}$) source is transmitted and polarized circularly after passing through a linear polarizer (90°) and quarter waveplate (45°). The incident light directed to an OI through a non-polarized beam splitter and propagated to a glass substrate was reflected from the ITO layer of the indicator due to specular reflection. Finally, passing through an analyzer the intensity of light is registered by a CCD camera^4,26^.

The visualization mechanism is based on the following phenomena. When the microwave is applied to the ITO glass, the alternating magnetic field of the microwave generates a surface current in the ITO layer. Such currents will heat the conductive layer due to Joule heating process caused by resistive losses of the ITO. The generated heat diffuses to the glass substrate and increases the local temperature of the glass. As a result, the circularly polarized light illuminating the indicator becomes elliptically polarized after specular reflection from the ITO layer due to a mechanically stressed medium (photoelastic effect). The stress distribution of the OI can be calculated by monitoring a change of the linear birefringence (LB) of the photoelastic medium. The CCD camera registers the elliptically polarized light with two different analyzer orientations: 0° and 45°. Stress analysis then shows the thermal distribution of the glass substrate caused by the microwave absorption of the ITO layer. The temperature of the thermoelastic medium can be calculated by solving the inverse problem of the mechanical stress distribution by using two images representing the results of linear birefringence. The calculation can be done by the following equation:

1 $$\frac{Q(x,y)}{C}=2\frac{{\partial }^{2}{\beta }_{2}(x,y)}{\partial x\partial y}+\frac{{\partial }^{2}{\beta }_{1}(x,y)}{\partial {x}^{2}}-\frac{{\partial }^{2}{\beta }_{1}\left(x,y\right)}{\partial {y}^{2}},$$

where $Q$ is the heat distribution, $C$ is the constant parameter correlated to the physical properties of the OI and related to the wavelength of the incident light. Supplementary Information and Ref.^26^ present more detailed information about the TEOIM system, including the actual photos.

When the electromagnetic signal is applied to the DUT, a specific frequency-dependent distribution of the electromagnetic field is generated in the vicinity of the DUT related to the geometrical characteristics of the filter. Depending on the electromagnetic and geometrical properties of the conductive layer of the OI, the visualized heat distribution will be related to either the electric or magnetic field distributions. In general, for non-magnetic materials, microwave heating is conditioned by two primary lossy mechanisms: dielectric and resistive losses. Resistive losses dominate in metallic and high conductive materials, whereas dielectric or dipolar losses dominate in dielectric insulators^28,29^. The dielectric loss is related to the electrical energy dissipation of insulating material by the oscillating electric field, and resistive loss is related to Joule heating of a highly conductive material by electrical currents induced by the oscillating magnetic field. Not extremely thin (above the electrical percolation threshold^30^) high conductive materials (Al, Au, Pt) deposited on the glass substrate will have a uniform surface and continuous conductivity along the surface. As a good conductor film, this type of metallic layer interacts with the microwave magnetic field and heats according to the magnetic field distribution. Otherwise, when the metallic film thickness is of the order of a few nanometers (below the electrical percolation threshold), the surface conductivity is intermittent because the surface of the metallic layer is spotty and composed of metallic nano-islands. In such dielectric layers, heating occurs due to dielectric losses under microwave irradiation. Metal nanoparticles embedded in glass substrates essentially increase the dielectric losses of material^31^. Thus, it is straightforward to find a proper coating in the case of the magnetic field indicator since it only requires a thin metallic layer with relatively high conductivity^26^. For instance, commercially available glass substrates covered by ITO layers with a thickness of several tens of nanometers (electrical conductivity of the order of ~ 10^5^–10^6^ S/m)^32^ can be exploited to visualize the magnetic fields. Moreover, simple aluminum or platinum mirrors also can be used for magnetic field visualization. However, finding an indicator is challenging in the case of electric field visualization. Previously published papers introduced the OI having high dielectric losses for electric-field visualization^26^. Those indicators are composed of XG glass substrate deposited with 5-nm-thin aluminum nano-islands on the surface and then covered by a PMMA thin film using a conventional spin coating technique. However, this preparation method involves several steps and is quite tricky, and the electromagnetic properties of the indicator sensitively depend on the layers' preparation conditions. During this process, several factors such as aluminum deposition rate, the size of nanoparticles, oxidation process in the room conditions, PMMA solution concentration, the spin coating speed, and the crystallization temperature can affect sample properties. Therefore, achieving the same electromagnetic properties for indicators is challenging after these complex preparation processes. This paper presents an easy method to make very sensitive and efficient electric field indicators based on the metasurfaces to avoid the aforementioned complex processes. For preparing the metasurfaces, commercially available uniform ITO glass was patterned by a straightforward laser ablation technique. Indicators made by excimer laser make it possible to achieve the same electromagnetic properties for each produced indicator every time, which is one of the advantages of using patterned indicators. Furthermore, the suggested indicators allow for separate detection of either |*E*_x_| or |*E*_y_| components of the electric field depending on the orientation of the pattern of the metasurface, whereas previously suggested nanoparticle-based electric field indicators could only visualize the in-plane component of the electric field distribution. The separate visualization of the electric field components using the metasurface-based indicators is the main novelty of this study. These materials can be included in single-target sensing applications, even in the biological environment.

## Simulation

**Figure 2:**
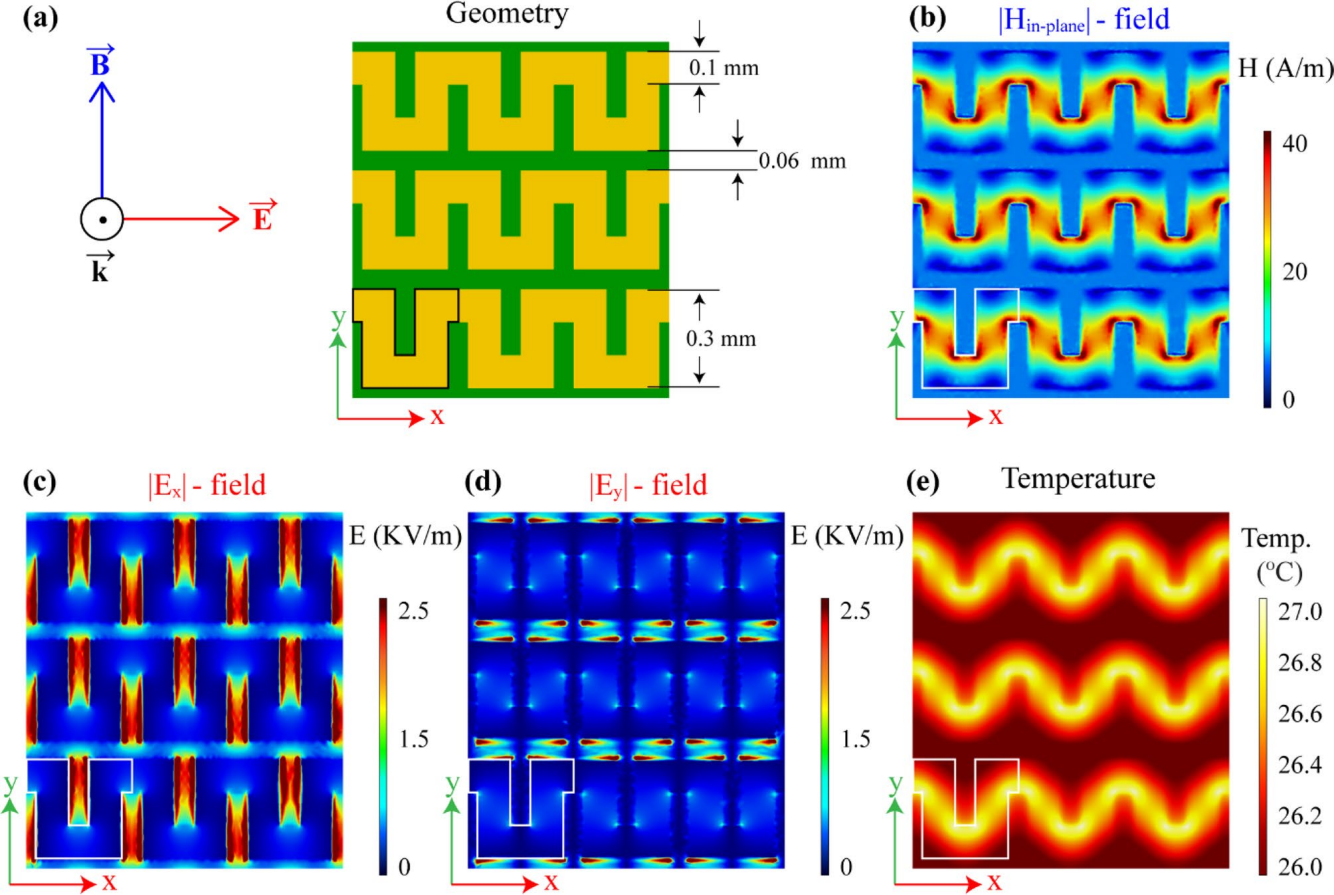
(**a**) Polarization direction of incident electromagnetic field and geometry of the metasurface structure. The simulation result of the indicator at 5 GHz for (**b**) in-plane magnetic field distribution (|*H*_in-plane_|), (**c**) *x*-component of electric field distribution (|*E*_x_|), and (**d**) *y*-component of electric field distribution (|*E*_y_|). (**e**) Thermal distribution on the metasurface under microwave radiation. Highlighted structures show the unit cell of the metasurface.

The working principle of the visualization system is based on the phenomenon of thermoelasticity. Under microwave irradiation, the metallic layer of the indicator strongly interacts with the electromagnetic field, and the heat distribution is generated on the surface of the OI. Therefore, the simulation model was designed for a proper investigation to understand the microwave heating behavior of the patterned indicators. Numerical simulations were carried out using the finite element method (FEM)-based COMSOL Multiphysics software. The geometry of the periodic structure is shown in Fig. 2a. Since the suggested method visualizes the electric and magnetic field distributions via the heat distribution caused by the dielectric or resistive losses in the patterned thin conductive structure, the “Heat Transfer in Solids” module combined with the “Electromagnetic Waves, Frequency Domain” module was used to simulate the thermal effects in the model. In the simulations, the result shows that the electric and magnetic field distributions are quite different on the surface of the designed structure. Numerical analyses were conducted to show the structure’s electric and magnetic field distributions under normal incident *x*- and *y*-polarized plane waves. The model of the periodic structure was simulated by applying periodic boundary conditions on the model walls. The Supplementary Information contains more details about simulation models and results.

In this structure, the continuity of the electrical conductivity was roughly split in the *y*-direction by a gap of 0.06 mm width. On the other hand, the effective conductivity was decreased compared with the continuous-phase film by introducing rectangular grooves in the *x*-direction (Fig. 2a). Since along the *x*-direction, the meander line is continuous, the grooves with sizes of 0.06 mm × 0.2 mm can be considered as a way to reduce the effective conductivity of the line in accordance with the surface fraction of the conductor^33^. Lowering the effective conductivity gives rise to the relatively improved coupling between the interacting microwave electric field and metasurface. This metasurface does not interact with the microwave magnetic field since the magnetic field can not generate the surface currents and heat the indicator by resistive losses because of the split surface conductivity. Thus, the indicator can be heated only due to the microwave electric field interaction. During the interaction of the incident microwaves with the pattern of the OI, a large current flow through the meander line is induced only when the incident electromagnetic field is polarized along the meander line, and in this case, the system is characterized by the inductive impedance corresponding to about 30% of the absorption of the incident microwaves and hence to the heating of the metasurface^34^. On the other hand, the incident electric field polarized perpendicular to the meander line does not induce any current flow and thus no metasurface heating is produced. Note that although the matching condition could be further improved by designing meander patterns having smaller surface fractions, such a geometry is chosen to ensure high enough intensity of the reflected light from the metasurface as the TEOIM uses the reflected light from the OI to visualize the electric/magnetic field distributions of the DUT. The microwave heating due to an electric field now dominates over the one caused by a magnetic field, and this OI will act as an electric field indicator. Because of the structural asymmetry the considered metasurface-based indicator will show the polarization sensitivity property under the incident electromagnetic field. For the incident field with a polarization state parallel to the meander line, the grooves of the meander conductive lines behave like capacitors and electromagnetic fields are localized between the gaps^35–37^. Moreover, the meander line acts as an inductive element.

The simulation results in Fig. 2 show the electric and magnetic field distributions on the metasurface under electromagnetic radiation when the polarization of the incident electromagnetic field corresponds to directions of meander lines (Fig. 2a). The in-plane component of the field is defined as

2 $${A}_{in{\text{-}}plane}=\sqrt{{{|A}_{x}|}^{2}+{{|A}_{y}|}^{2}},$$

where *A* denotes the amplitude of the electric *E-* or magnetic *H-* field. Figure 2b shows the in-plane magnetic field distribution on the surface of the periodic structure. Figure 2c,d depict the magnitudes of the electric field |*E*_x_| and |*E*_y_| components, respectively, under the *x*-polarized plane wave. One sees that a strong electric field is concentrated between the meander grooves, which act as capacitors for the incident field polarized along the groove width. Numerical analysis shows that due to the large charge density around the sharp edges of the grooves, the electric field is stronger in this local region. Thus, the coupling between the incident microwave field and the ITO metasurface is stronger. Due to this coupling effect, the metallic surface heats up, and the heat distribution is shown in Fig. 2e. The result shows that the heat distributed along the metallic lines of the metasurface is similar to a magnetic in-plane field distribution shown in Fig. 2b. It appears that in the metasurface, the heat was generated via a magnetic field, but microwave coupling between the metasurface and the incident microwave field occurs only when the polarization direction of the incident wave corresponds to the direction of the meander lines. After rotating the incident microwave polarization by 90°, the metasurface does not show any coupling effect with the microwave field. The simulation result in the case when the polarization of the incident microwave field is perpendicular to a meander line is shown in the Supplementary Information Sect. 2. In that configuration, the metasurface does not show noticeable temperature changes under microwave radiation. The reason for this behavior is a polarization-sensitive property of the designed metasurface, which means that for a normal incident *y*-polarized plane wave, the metasurface is mainly transparent.

## Results and discussion

**Figure 3:**
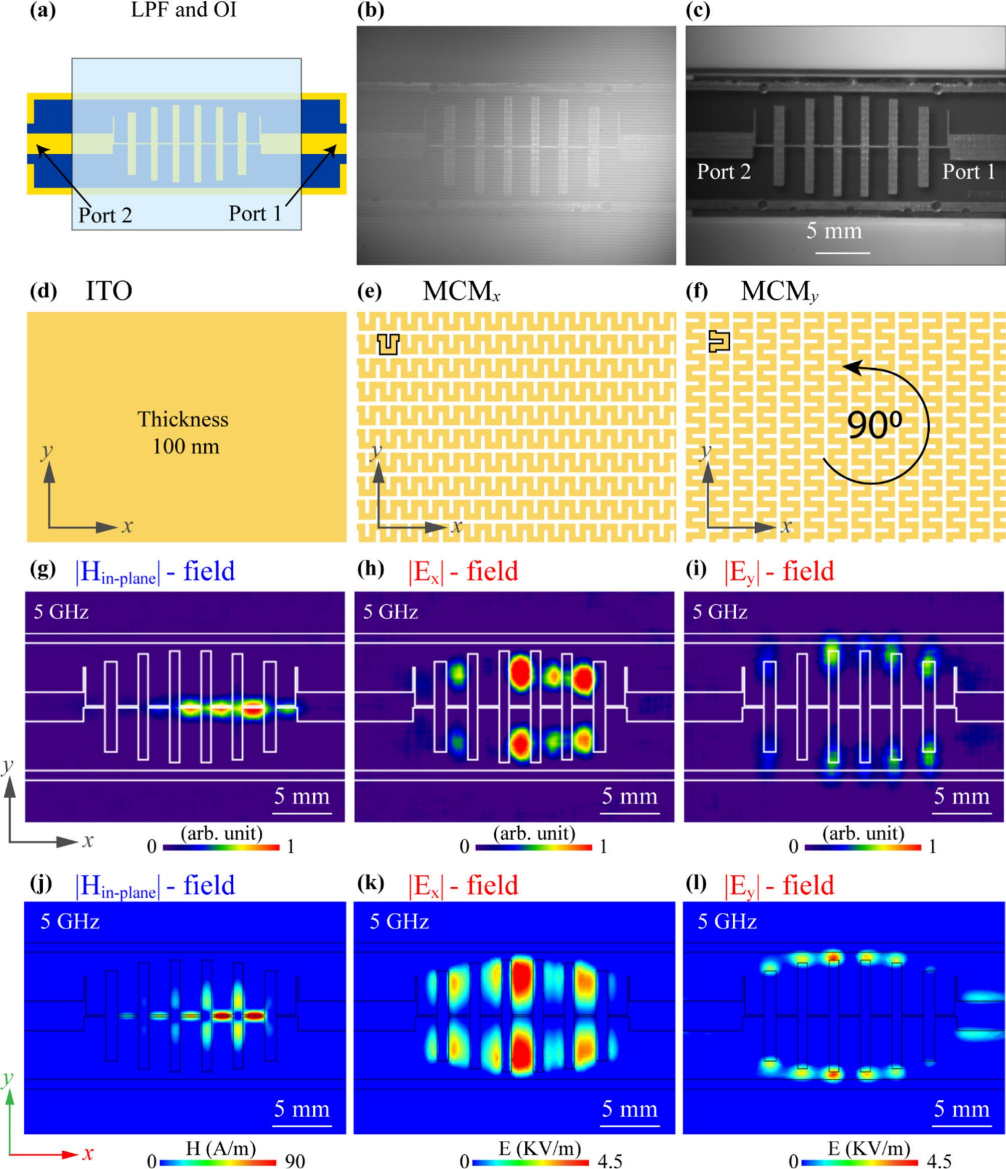
(**a**) Illustration of the LPF under test. Optical image of DUT (**b**) with and (**c**) without indicator captured by CCD camera. (**d**) Illustration of the uniform ITO glass indicator. Patterned ITO glass metasurface for (**e**) MCM_x_ and (**f**) MCM_y_. Visualized result of MWNF distribution using (**g**) ITO glass, (**h**) MCM_x_-metasurface, and (**i**) MCM_y_-metasurface at 5 GHz. Simulation result of (**j**) in-plane magnetic field distribution (|*H*_in-plane_|), (**k**) *x*-component of electric field distribution (|*E*_x_|), and (**l**) *y*-component of electric field distribution (|*E*_y_|) at 5 GHz.

Figure 3 shows the OI structure and the results of the MWNF distributions of the measurement and simulation. To demonstrate that the designed OI can be used for the selective visualization of the electric field *x*- and *y*-components, a simple stepped impedance low-pass filter (LPF) was chosen as a DUT whose schematic sketch is shown in Fig. 3a. This filter structure is appropriate for indicator analysis since the distributions of the magnetic field, and the *x*- and *y*-components of the electric field are noticeably different; making it easy to explore the operation principles of the suggested OI. The graph of *S*-parameters of the DUT is included in the Supplementary Information Sect. 3. The field distributions are visualized by placing the corresponding OI on the surface of the DUT with a small air gap. Figure 3b,c are the optical images of the DUT (top view) with and without the indicator. The microwave absorption and thus heating mechanisms can be different depending on the material and structural features of the OI. When the OI is composed of a uniform metallic thin film, surface currents induced by the magnetic field are responsible for the heating of the indicator^38,39^.

Figure 3d–f show the schematics of the continuous ITO film, structures of the patterned MCM when the meander lines oriented along the *x*-axis (denoted MCM_x_) and the *y*-axis (denoted MCM_y_), respectively. Figure 3g shows the magnetic field distribution of LPF at 5 GHz, where uniform ITO glass was used as an OI. The patterned indicator with the proper orientation can visualize only one component of the electric field distribution of the RF device. The |*E*_x_| component of the electric field (Fig. 3h) was visualized by using the OI illustrated in Fig. 3e. Likewise, Fig. 3i depicts the distribution of the electric field |*E*_y_| component visualized with the OI shown in Fig. 3f. The operating frequency was 5 GHz for all cases. These experimental results are strongly confirmed by the full-wave numerical simulations based on the FEM shown in Fig. 3j–l representing the simulated distributions of the |*H*_in-plane_|, |*E*_x_| and |*E*_y_| components of the electromagnetic field of the DUT. The experimental and simulation results show that the magnetic field is mainly localized around the high impedance line (HIL), while the electric field is localized between the vertical lines crossing the HIL, far from the HIL. In particular, for the *y*-component of electric field distribution, the energy is localized only on the ends of the vertical lines. One of the main advantages of the designed OI is the ability to visualize only one component of the electric field, and due to the polarization sensitivity of the OI, the same indicator can be simply rotated by 90° and used to visualize the other component of the electric field, thus distinguishing each part of the E-field.

**Figure 4:**
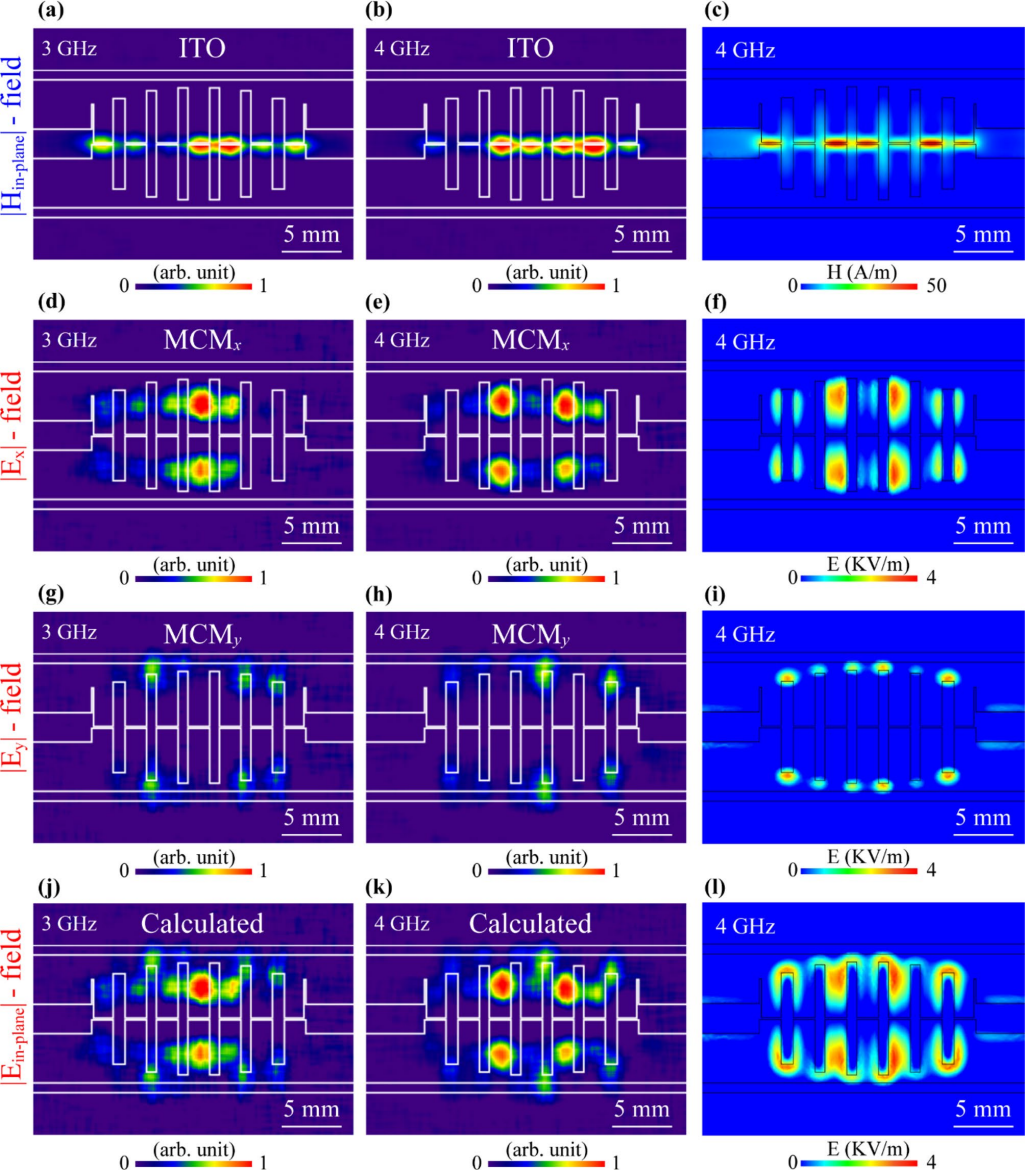
Visualized and simulated distributions of the electric and magnetic fields of LPF. The first row represents the visualized and simulated results for in-plane magnetic field distributions (|*H*_in-plane_|) using ITO glass indicator at (**a**) 3 GHz and (**b**) 4 GHz. (**c**) Simulation result corresponding to (**b**). The second row represents the visualized and simulated results for the *x*-component of electric field distribution (|*E*_x_|) using MCM_x_-metasurface at (**d**) 3 GHz and (**e**) 4 GHz. (**f**) Simulation result corresponding to (**e**). The third row represents the visualized and simulated results for the *y*-component of electric field distribution (|*E*_y_|) using MCM_y_-metasurface at (**g**) 3 GHz and (**h**) 4 GHz. (**i**) Simulation result corresponding to (**h**). The fourth row represents the calculated and simulated results for the in-plane electric field distribution (|*E*_in-plane_|) at (**j**) 3 GHz and (**k**) 4 GHz. (**l**) Simulation result corresponding to (**k**).

Using the same DUT, electric and magnetic field distributions at 3 GHz and 4 GHz were visualized to show that designed OI can properly operate in different frequency ranges. Moreover, the experimental results at 4 GHz were compared to the corresponding simulation results. Figure 4a,b show visualized distributions of the in-plane magnetic field component at 3 GHz and 4 GHz, respectively, and Fig. 4c shows the distribution of the same component for 4 GHz obtained via FEM-based simulations. Keeping the same manner of presentation, Fig. 4d–i depict the visualized (at 3 GHz and 4 GHz) and simulated (at 4 GHz) distributions of the electric field |*E*_x_| and |*E*_y_| components, respectively. Although the designed indicator visualizes only one electric field component, it is possible to retrieve the in-plane electric field distribution via simple image-processing according to Eq. (2) by using experimentally obtained distributions of the electric field *x*- and *y*-components. For instance, Fig. 4j shows the calculated in-plane electric field distribution by using the images shown in Fig. 4d,g at 3 GHz. By the same method, in Fig. 4k in-plane electric field distribution was calculated by using images shown in Fig. 4e,h at 4 GHz.

**Figure 5:**
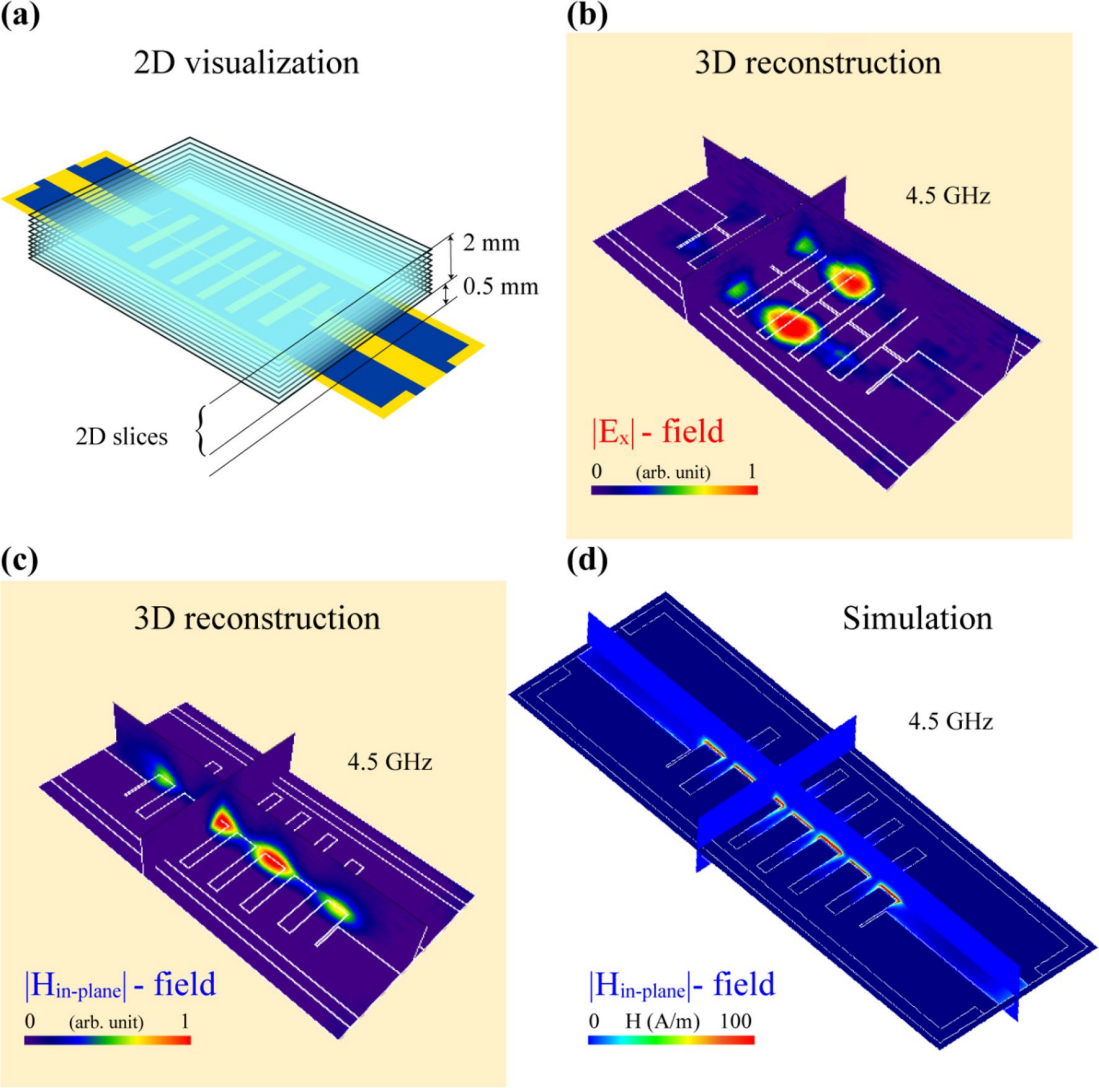
(**a**) Illustration of the LPF under test with the OI placed at different locations from metasurface with the various measurement steps. 3D reconstruction of the LPF at 4.5 GHz for (**b**) *x*-component of electric field distribution (|*E*_x_|) and (**c**) in-plane magnetic field distribution (|*H*_in-plane_|). (**d**) Simulation result for in-plane magnetic field distribution.

These electric field in-plane distributions obtained at 4 GHz (Fig. 4k) were compared to simulation results (Fig. 4l) and are in good agreement. Besides the LPF, another filter structure was tested via a TEOIM system using the same methods and indicators as above. The results of the electric and magnetic field visualizations of a hairpin band-pass filter (BPF) with a different structural shape compared with the LPF are included in Supplementary Material Sect. 4. In both cases, the designed indicators show comparable results for field distributions obtained by a numerical analysis. The fundamental understanding of the microwave heating phenomenon of the designed indicators is relatively complex. It is challenging to theoretically represent the heating principles under the microwave irradiation because of the features and complex coupling behavior of the electromagnetic wave with a patterned thin metallic film when the thickness of the metal is very small compared with the skin depth^28^. Computer modeling of such a system, as in this case, is an excellent method to obtain a full representation of the principles of microwave heating. This is the main reason for comparing the experimental result with the simulation models and this method of comparison is self-sufficient. Indicators were also tested in practical applications involving the visualization of RF filters. In particular, the designed OI can be exploited to scan and visualize the spatial distribution of the electromagnetic fields in the region above the DUT surface where the radiated field source is present. To achieve this, the distance of the indicator and the DUT was sequentially increased. The illustration in Fig. 5a shows the structure of LPF and the OI placed in the near region of the device with different positions after each visualization step. The first visualized distribution of the electric field was obtained with the air gap thickness between the OI and DUT of 0.5 mm. This air gap prevents changes of scattering parameters of the RF filter. Therefore, starting from this position, the distance was increased by 0.1 mm after each imaging step, and the process continued until the measurement did not show any field distribution. It is a scanning process through the *z*-axis, where 2D slices of the MWNF distribution images were collected, and intensities were normalized together. Experimental and simulation results show that microwave power is localized only in the nearest regions of the microstrip lines. After moving the indicator by a total 2.5 mm from the DUT surface, twenty images were obtained for further image processing. The step size of this scanning process is negligibly small compared to the wavelength of the operating frequency, which allows us to make a simple mathematical approximation between two neighbor slices without any roughness. The image analysis software imageJ^40^ was used to reconstruct a 3D bulk image of the MWNF distribution using a visualized 2D stack.

Figure 5b depicts the 3D reconstructed image of the *x*-component of electric field distribution with two orthoslices crossing the *x*- and *y*-axis where the actual size of the *z*-axis extension is 2 mm. Figure 5c shows the in-plane magnetic field distribution of the LPF, and for comparison, the simulation result is included in Fig. 5d. The measurement and simulation results show that in the case of RF filters, the microwave power is distributed only in the vicinity of the filter surface. The Supplementary Video 1 sequentially shows the 360° rotation around 3D reconstructed field distributions of the in-plane magnetic field, *x*-and *y*-components of the electric field for LPF and BPF. This 3D reconstruction method can be a great practical tool for the investigation and optimization purposes of patch antennas by visualizing the radiation patterns of the designed structure^3^. TEOIM system can be an alternative to computer simulation programs allowing one to bring the investigation to a real-condition experimental environment. Also, TEOIM is an excellent tool to visualize MWNF distribution on the surface of metamaterials which can be helpful for the design optimization and thorough understanding of physical processes related to the interaction of the incident field and metamaterial unit cell. Even if the metamaterial structure is designed directly on the transparent and thermoelastic material, such as a glass substrate, it can be simultaneously used as the OI and DUT. The role of transparent conductive oxides and particularly the ITO-based sensors increases over time, and due to its unique properties, this material is a promising candidate to develop sensors for medical and technological applications^41–43^. The modification of the ITO film opens the new physical and sensing properties of this material and increase the area of interest as presented above. The suggested structure does not limit the visualization ability of the TEOIM. Apparently, it is possible to reach a higher visualization resolution and sensitivity by decreasing the cell size of the structure. Moreover, using the ideas presented in this work, new metasurface-based indicators with more efficient and improved visualization properties can be designed.

## Conclusions

A new periodic structure was designed on the basis of ITO glass, functioning as an OI for the TEOIM system. It was shown that the designed metasurface-based OI can be used to visualize either *x-* and *y-* components of the electric field distribution. The indicator was tested to visualize field distributions of two different RF filters at several operating frequencies demonstrating a good agreement with the corresponding numerical simulations. Starting from the near region of the RF filter, the spatial distribution of the MWNF was visualized by changing the distance between the OI and RF filter, where all 2D images were collected for further 3D field reconstruction. The reconstructed 3D spatial distributions can be used for the investigation of various interference phenomena. Finally, possible opportunities were discussed for the TEOIM system with advanced OI.

## Supplementary Information

Supplementary Information.

Supplementary Video 1.
