# Supplementary material for: 3D visualization of microwave electric and magnetic fields by using a metasurface-based indicator
Source: Sci Rep. 2022 Apr 12;12:6150. doi: 10.1038/s41598-022-10073-7 (PMC9005508; doi:10.1038/s41598-022-10073-7)
Supplement: Supplementary file 1 — Supplementary Information. [file 41598_2022_10073_MOESM1_ESM.docx]

**Supplementary information**

**3D visualization of microwave electric and magnetic fields by using a metasurface-based indicator**

Zhirayr Baghdasaryan^1,2^, Arsen Babajanyan^2^, Henrik Parsamyan^2^, Barry Friedman^3^, Seungwan Kim^1^, Jung-Ha Lee^4^, Kiejin Lee^1,*^

*^1^Department of Physics, Sogang University, Seoul 121-742, Korea*

*^2^Department of Radiophysics, Yerevan State University, Yerevan 0025, Armenia*

*^3^Department of Physics, Sam Houston State University, Huntsville, Texas 77341, USA*

*^4^Department of Life Science, Sogang University, Seoul 121-742, Korea*

**Corresponding Author: Email:* [*klee@sogang.ac.kr*](mailto:klee@sogang.ac.kr)

**Supplementary video 1.** 360-degree rotation around 3D reconstructed field distributions of the in-plane magnetic field, *x*-component and *y*-component of electric field for LPF and BPF.

**Supplementary Information 1**

Figure S1 (a) shows the real configuration of the TEOIM visualization system. All measurements were implemented in the dark environment to prevent alternative light noises at room temperature. The microwave heating emerging in the glass substrates is very local related to a field distribution, and variation of the room temperature has no significant effect on the measurement processes, which was ignored during the experiments. Figure S1 (b) shows the experimental adjustment for DUT with an OI, where the distance of the OI and DUT is 0.5 mm, which is the minimal distance where the metallic layer of the OI has a minimal effect on the transmission property of the RF filter. During the stationary MWNF imaging, the particular field distribution is obtained by subtracting two averaged images of the sample with and without applied electromagnetic (EM) waves. Due to the process, any alternative influence of the ambient temperature vanishes. These averaging and smoothing steps help reduce the errors related to the defects of the indicator and possible environmental instability.

**
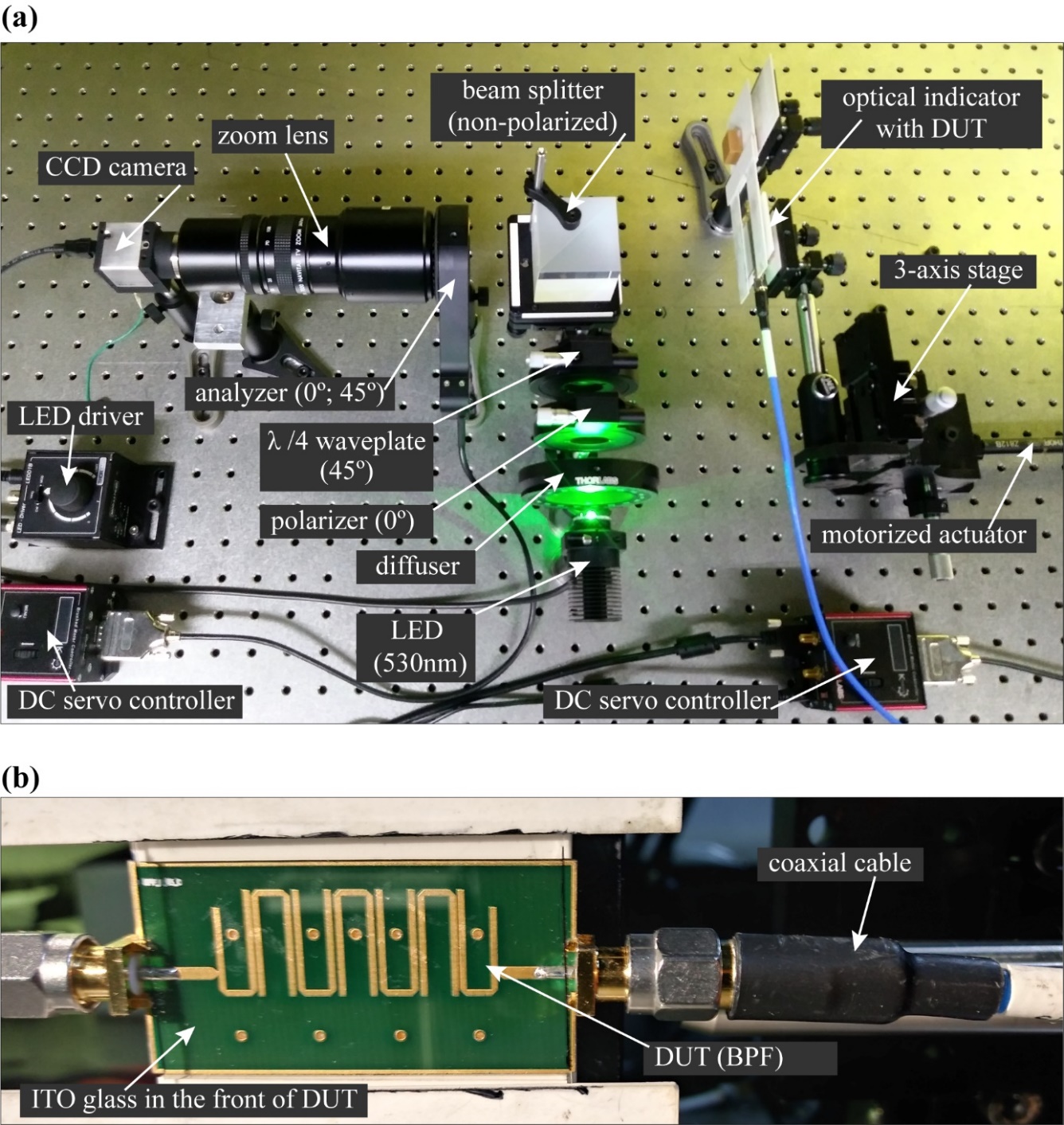
**

**Supplementary figure S1.** (**a**) The photograph of the TEOIM visualization system and (**b**) experimental adjustment for DUT.

**Supplementary Information 2**

The indicator is composed of a patterned ITO film with a thickness of 100 nm. The simulation model consists of two physics interfaces – *“*Electromagnetic Waves, Frequency Domain*”* and *“*Heat Transfer in Solids*”*. Since the suggested method visualizes the electric field distribution via the heat distribution caused by the resistive or dielectric losses in the thin conductive patterned structure^1^, the *“*Heat Transfer in Solids*”* module combined with the EM field module was used to simulate the thermal effects in the model. Numerical analyses were also conducted to show the electric and magnetic field distributions of the structure under normal incident x- and y-polarized plane waves. The model of the periodic structure is simulated by applying periodic boundary conditions on the model walls.

| **Property** | **Symbol** | **Value** |
| --- | --- | --- |
| Thickness | *d* | 100 (nm) |
| Density | *ρ* | 7120 (kg/m^3^) |
| Thermal conductivity | *K* | 4 W/(m·K) |
| Specific heat capacity | *C_p_* | 341 J/(kg·K) |
| Electrical conductivity | *σ* | 1.25×10^6^ (S/m) |

**Supplementary table 1.** Characteristic parameters used in simulations for modeling ITO layer^2^.


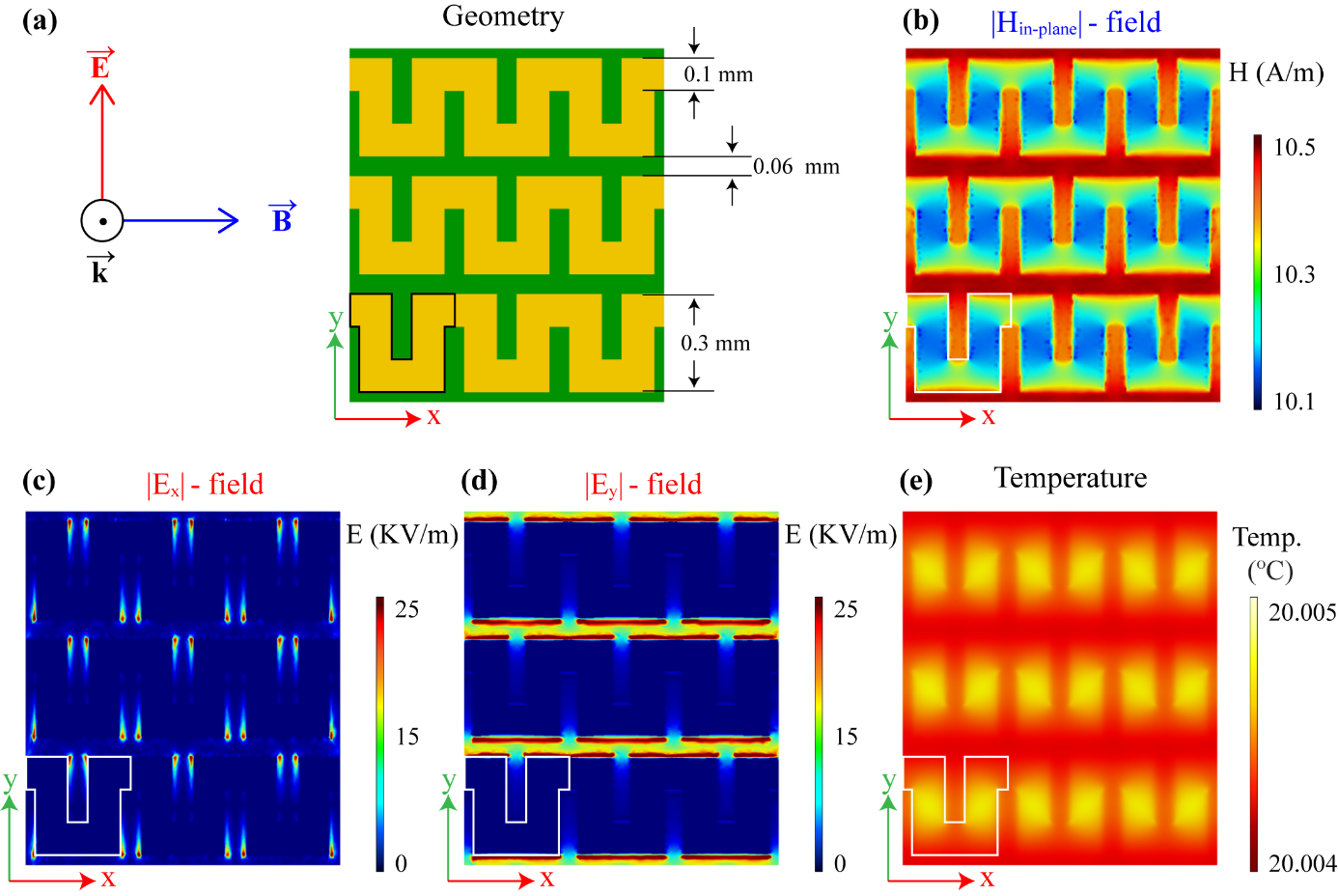


**Supplementary figure S2.** (**a**) Polarization direction of incident electromagnetic field and geometry of the metasurface structure. The simulation result of the indicator at 5 GHz for (**b**) in-plane magnetic field distribution (|*H*_in-plane_|), (**c**) *x*-component of electric field distribution (|*E*_x_|), and (**d**) *y*-component of electric field distribution (|*E*_y_|). (**e**) Thermal distribution on the metasurface under microwave radiation. Highlighted structures show the unit cell of the metasurface.

**Supplementary Information 3**

The


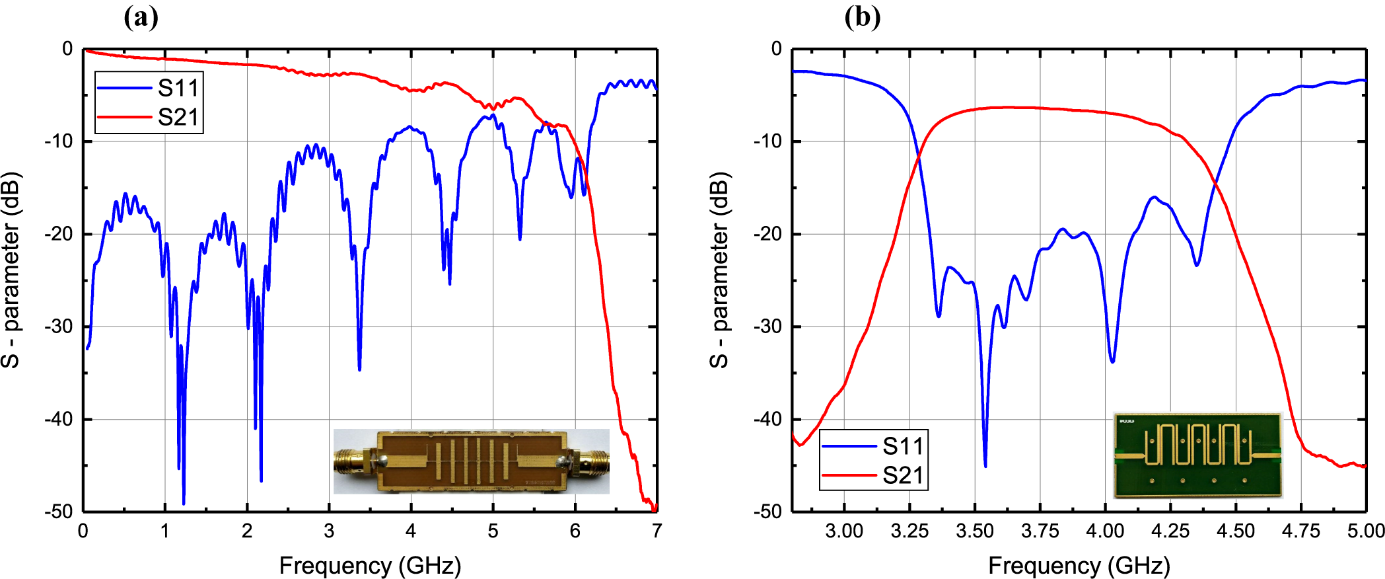


**Supplementary figure S3.** *S*-parameters of the (**a**) LPF and (**b**) BPF.

**Supplementary Information 4**


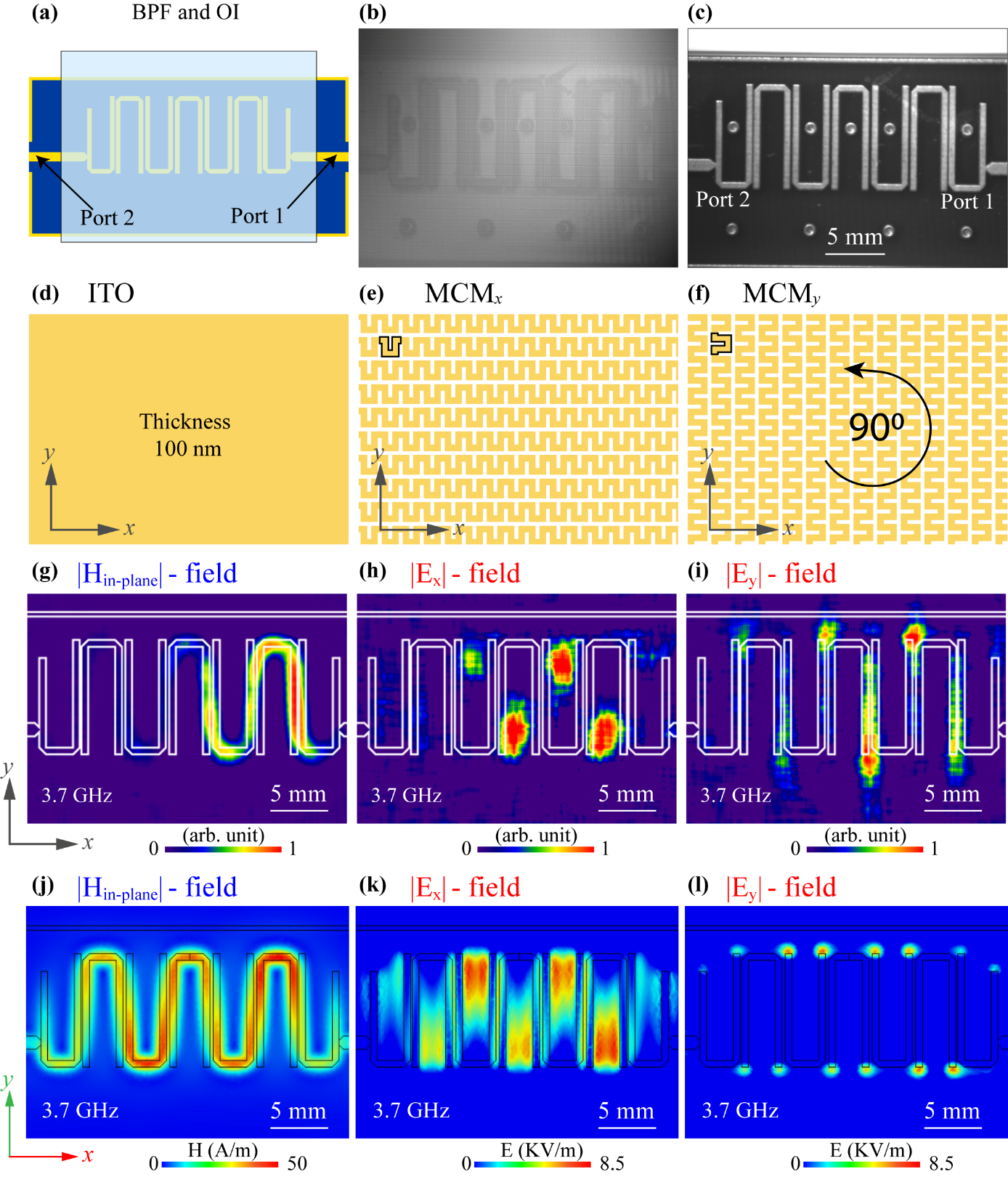


**Supplementary figure S4.** (**a**) Illustration of the BPF under test. Optical image of DUT (**b**) with and (**c**) without indicator captured by CCD camera. (**d**) Illustration of the uniform ITO glass indicator. Patterned ITO glass metasurface for (**e**) MCM_x_ and (**f**) MCM_y_. Visualized result of MWNF distribution using (**g**) ITO glass, (**h**) MCM_x_-metasurface, and (**i**) MCM_y_-metasurface at 3.7 GHz. Simulation result of (**j**) in-plane magnetic field distributions (|*H*_in-plane_|), (**k**) *x*-component of electric field distribution (|*E*_x_|), and (**l**) *y*-component of electric field distribution (|*E*_y_|) at 3.7 GHz.


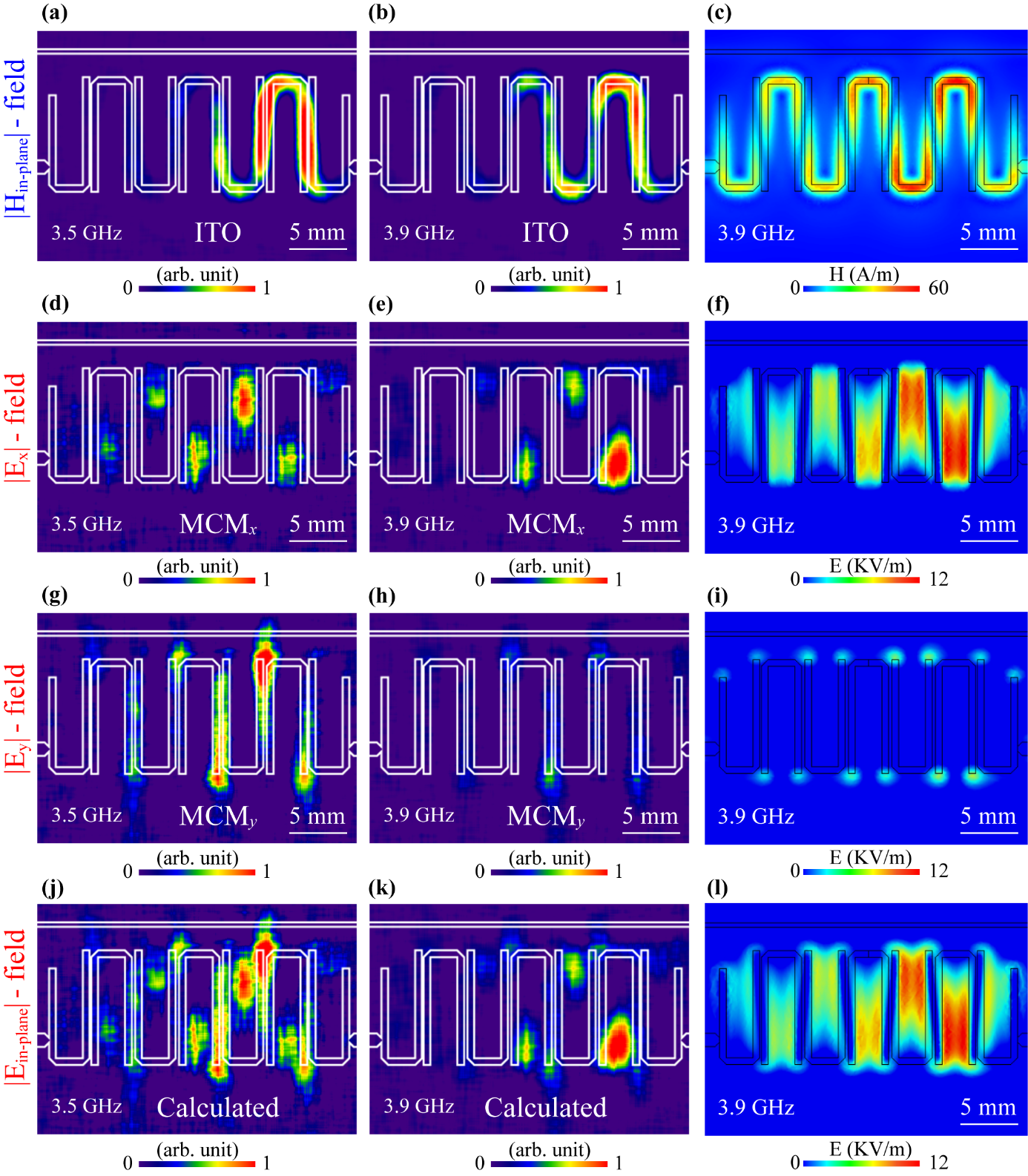


**Supplementary figure S5.** Visualized and simulated distributions of the electric and magnetic fields of BPF. The first row represents the visualized and simulated results for in-plane magnetic field distribution (|*H*_in-plane_|) using ITO glass indicator at (**a**) 3.5 GHz and (**b**) 3.9 GHz. (**c**) Simulation result corresponding to (**b**). The second row represents the visualized and simulated results for the x-component of electric field distribution**(**|*E*_x_|) using MCM_x_-metasurface at (**d**) 3.5 GHz and (**e**) 3.9 GHz. (**f**) Simulation result corresponding to (**e**). The third row represents the visualized and simulated results for the y-component of electric field distribution**(**|*E*_y_|) using MCM_y_-metasurface at (**g**) 3.5 GHz and (**h**) 3.9 GHz. (**i**) Simulation result corresponding to (**h**). The fourth row represents the calculated and simulated results for the in-plane electric field distribution (|*E*_in-plane_|) at (**j**) 3.5 GHz and (**k**) 3.9 GHz. (**l**) Simulation result corresponding to (**k**).

**REFERENCES**

1. Lee, H., Arakelyan, S., Friedman, B. & Lee, K. Temperature and microwave near field imaging by thermo-elastic optical indicator microscopy. *Sci. Rep.* **6**, 1–11 (2016).

2. Wang, L., Wen, J., Yang, C. & Xiong, B. Potential of ITO thin film for electrical probe memory applications. *Sci. Technol. Adv. Mater.* **19**, 791–801 (2018).
